# Make it SIMPLE: enhanced shock management by focused cardiac ultrasound

**DOI:** 10.1186/s40560-016-0176-x

**Published:** 2016-08-15

**Authors:** Ka Leung Mok

**Affiliations:** Accident and Emergency Department, Ruttonjee Hospital, 266 Queen’s Road East, Wanchai, Hong Kong SAR

**Keywords:** Shock, Ultrasound, Echocardiography, Emergency department, Critical care, Sepsis

## Abstract

**Background:**

Shock is a spectrum of circulatory failure that, if not properly managed, would lead to high mortality. Special diagnostic and treatment strategies are essential to save lives. However, clinical and laboratory findings are always non-specific, resulting in clinical dilemmas.

**Main content:**

Focused cardiac ultrasound (FoCUS) has emerged as one of the power tools for clinicians to answer simple clinical questions and guide subsequent management in hypotensive patients. This article will review the development and utility of FoCUS in different types of shock. The sonographic features and ultrasound enhanced management of hypotensive patients by a de novo “SIMPLE” approach will be described. Current evidence on FoCUS will also be reviewed.

**Conclusion:**

Focused cardiac ultrasound provides timely and valuable information for the evaluation of shock. It helps to improve the diagnostic accuracy, narrow the possible differential diagnoses, and guide specific management. SIMPLE is an easy-to-remember mnemonic for non-cardiologists or novice clinical sonographers to apply FoCUS and interpret the specific sonographic findings when evaluating patients in shock.

## Background

Shock is a clinical syndrome in which there is inadequate cellular and tissue oxygenation due to circulatory failure [1]. The presentation of shock can vary with different causes of shocks and degrees of physiological abnormalities. Shock can be classified into five different categories according to the underlying pathophysiology, namely hypovolemic shock (due to hemorrhage or intravascular volume depletion), cardiogenic shock (e.g., acute myocardial infarction, myocarditis), obstructive shock (e.g., pulmonary embolism, tension pneumothorax, and cardiac tamponade), and distributive shock (e.g., septic, neurogenic, and anaphylactic), and lastly, shock related to cellular poisoning [2]. One of the cardinal features of shock is hypotension. It can be defined as systolic blood pressure lower than 90 mmHg or more precisely mean arterial pressure lower than 65 mmHg as suggested by the latest international consensus definitions for sepsis and septic shock [3]. It is associated with high mortality and adverse hospital outcomes in non-traumatic patients in the emergency department [4, 5].

In order to save our patients in shock, early diagnosis, and timely targeted therapy is vital. To do so in a timely manner is a challenge as clinical presentation of different types of shock may be similar. Point-of-care ultrasound (PoCUS) performed by clinicians providing direct care to the patients is considered an invaluable clinical tool to facilitate diagnosis-making, to rule out potentially fatal conditions, and to provide guidance to life-saving procedures [6]. Among the different applications of PoCUS, focused cardiac ultrasound (FoCUS) is gaining popularity in emergency care settings. It is considered as one of the core emergency ultrasound applications by the American College of Emergency Physicians and the International Federation for Emergency Medicine [7, 8]. Recently, FoCUS has been integrated into scanning protocols together with focused scans in other regions, e.g., lung, abdomen, and lower limb deep vein system to manage patients in clinically undifferentiated hypotensive state [9–11]. In the following sessions, the SIMPLE approach, the role of FoCUS in the management of shock, and the current evidence for this application will be discussed.

### Essentials of FoCUS and SIMPLE approach

The name “focused cardiac ultrasound” (FoCUS) is interchangeable with “focused echocardiography,” “emergency echocardiography,” “bedside limited echocardiography,” “point-of-care cardiac ultrasound,” and “goal-directed echocardiography” [12]. Lately, the term “focused cardiac ultrasound” has been recognized as a more appropriate term to take into account the nature of point-of-care application of ultrasound assessment of cardiac anatomy and physiology, distinct from the formal echocardiographic study done by cardiologists, according to the first international evidence-based recommendations issued by World Interactive Network Focused on Critical UltraSound (WINFOCUS) [13]. FoCUS was first introduced into emergency communities in the 1990s [14, 15]. With the wider availability and miniaturization of ultrasound machines, FoCUS has quickly become standard practice in acute care settings across the globe. In contrast to the conventional comprehensive echocardiography performed in the cardiac laboratory by cardiologists, FoCUS is performed by emergency physicians or intensivists at the bedside. It is essentially a limited evaluation of cardiac function, pericardial space, and intravascular volume in order to answer clinical questions vital to patient management. Contrary to what some may believe, the requirement for the FoCUS is not high. A portable or even pocket-sized handheld ultrasound machine can provide adequate image quality for assessment of left ventricular function, detection of pericardial effusion, and measurement of abdominal aorta size [16–18]. Pocket-sized machines are advantageous in unfavorable environments where full-sized machines will be impractical, e.g., pre-hospital assessment in an ambulance or helicopter [19].

**Fig. 1:**
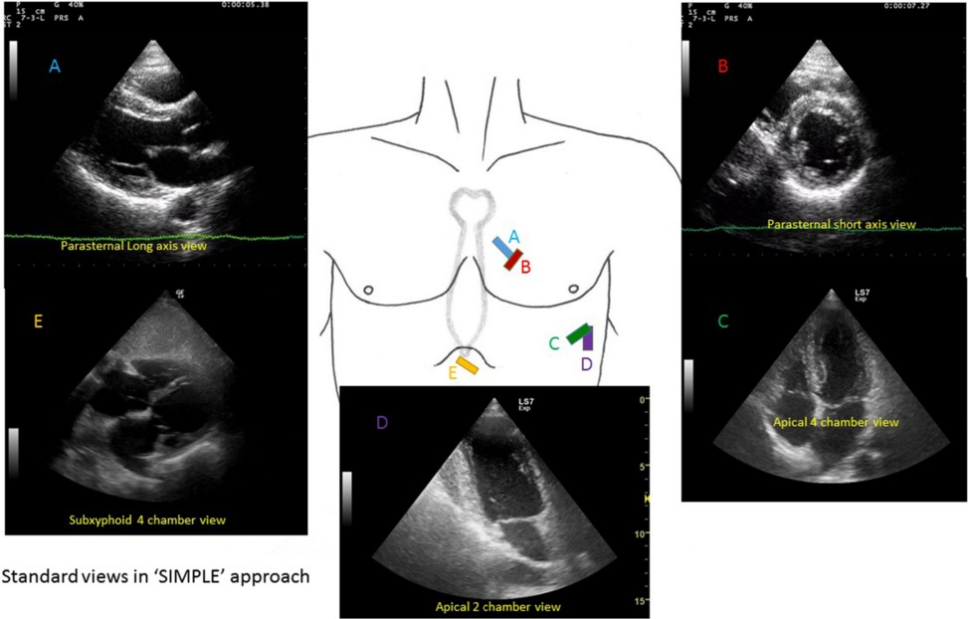
Five standard views used in focus echocardiography. They include parasternal long axis (**a**), parasternal short axis (**b**), apical four-chamber (**c**), apical two-chamber (**d**), and subxyphoid four-chamber views (**e**)

**Fig. 2:**
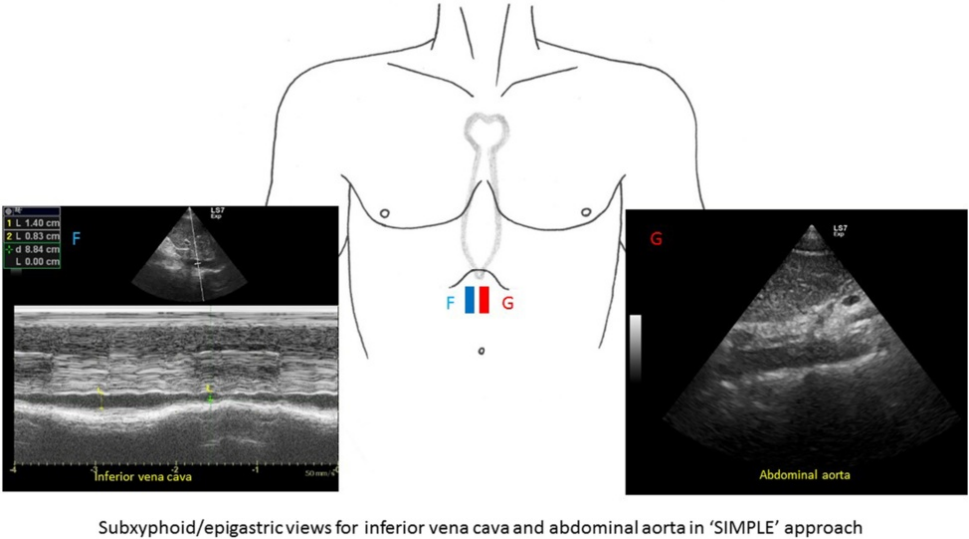
Subxyphoid/epigastric views for inferior vena cava (*F*) and abdominal aorta assessment (*G*)

FoCUS makes use of the same five orthodox views as in transthoracic echocardiographic study (TTE), (Fig. 1) to assess cardiac function, namely the left parasternal long and short axis views, apical four-chamber view, apical two-chamber view, and subxyphoid four-chamber view. Besides, subcostal visualization of the inferior vena cava (IVC) is frequently integrated into FoCUS to assess volume status and fluid responsiveness in hypotensive patients (Fig. 2) [9, 10]. 2D imaging and M-mode are employed for assessment in FoCUS. Doppler study is reserved for more sophisticated measurements in the cardiac laboratory, such as in assessing valvular dysfunction, calculating stroke volume, and mitral inflow velocity. These measurements would take longer time to achieve and may technically difficult during the initial phase of resuscitation when adequate visualization of the heart cannot be easily obtained. According to the international evidence-based recommendations for FoCUS, Doppler assessment of valvular dysfunction is considered beyond the scope of FoCUS and reserved for evaluation by standard comprehensive echocardiography [13]. Thus, this review will mainly focus on the application of 2D imaging and M-mode study to rapidly assess patients in shock.

In the assessment of hypotensive patients, several key sonographic findings should be evaluated. They include the chamber sizes, in particular the left ventricle (LV) and the right ventricle (RV), the interventricular septum (IVS), the IVC, the presence of intramural mass (commonly blood clots and myxoma), myocardial thickness and motion during systole, the presence of pericardial effusion or pleural effusion, LV systolic function and the abdominal aorta in the epigastrium. All of these can be summarized into a “SIMPLE” approach (Table 1). It will give emergency physicians and intensivists a useful checklist in the evaluation of hypotensive patients.

**Table 1:** SIMPLE approach for evaluation of key elements during focused cardiac ultrasound sound (FoCUS) in shock patients

SIMPLE approach in focused cardiac ultrasound	
*S*	Chamber *s*ize and *s*hape, particularly LV and RV *s*ize
*I*	*I*VC size and collapsibility *I*VS movement *I*ntimal flaps inside the aorta, suggestive of aortic dissection
*M*	*M*ass in the heart chambers (commonly intramural clots and atrial myxoma) *M*yocardium (motion and thickness)
*P*	*P*ericardial effusion *P*leural effusion
*L*	*L*eft ventricular systolic function
*E*	Abdominal aorta in the *e*pigastrium

**Table 2:** Summary of typical findings in different types/causes of shock by SIMPLE approach

Type of shock		Hypovolemic	Cardiogenic	Septic	Distributive	Pulmonary embolism	Cardiac Tamponade	Aortic Dissection
*S*	*Chamber size*	Small LV	Dilated LV	Early: small LVESA Late: normal/dilated	Near normal LVEDA but small LVESA	Dilated RV, small/normal LV	Diastolic collapse of RA and RV; normal LV	Usually normal
*I*	*IVC thickness*	Collapsed	Distended <50 % respiratory collapse	Early: collapsed Late: distended	Collapsed	Distended and loss of respiratory collapse	Distended and loss of respiratory collapse	Normal when no cardiac tamponade
	*IVS movement*	Normal	Reduced	Early: normal Late: reduced	Normal	Paradoxical IVS and D-shaped LV	Normal	Normal
	*Intimal flap*	Absent	Absent	Absent	Absent	Absent	Absent	Present
*M*	*Myocardial thickening/motion*	Hyperdynamic	Hypokinetic	Early: hyperdynamic Late: hypokinetic	Hyperdynamic or normal	McConell’s sign, LV hyperdynamic	Diastolic collapse of RA and RV	Normal if coronary ostia not involved
	*Masses in heart*	Absent	Intramural thrombi if AF/AMI	Absent	Absent	Thrombi in RA/RV and IVC	Absent	Absent
*P*	*Pericardial effusion*	Absent	Small amount if inflammatory cause	Absent	Absent	Absent	Moderate to large but can be small if acutely collected	Present if retrograde dissection and echogenic
	*Pleural effusion*	Absent	Present	Present if pneumonia	Absent	Usually absent	Absent	Present if hemothorax
*L*	*LV systolic function*	Hyperdynamic	Poor	Early: normal or hyperdynamic Late: impaired	Normal or hyperdynamic	Normal or hyperdynamic	Normal	Normal
*E*	*Abdominal aorta in epigastrium*	Aneurysmal if due to AAA rupture	Normal	Normal	Normal	Normal	Normal	Intimal flap seen

*AF* atrial fibrillation, *AMI* acute myocardial infarct, *LV* left ventricle, *LVEDA* left ventricular end-diastolic area, *LVESA* left ventricular end-systolic area, *RA* right atrium, *RV* right ventricle

In addition to the five orthodox TTE views, the subcostal region or epigastrium is included in this SIMPLE scanning protocol to assess the size of IVC and abdominal aorta which may be involved in aortic dissection and aneurysmal rupture (Fig. 2). In this approach, a single ultrasound probe is used to look for the causes for hypotension and guide treatment by means of a focused point-of-care ultrasound study. Concerning the sequence of examination, it would be a good habit to start at the parasternal views then move to the apical view and, finally, the subxyphoid/epigastric regions to assess the IVC and the abdominal aorta. However, in some patients with emphysematous lungs, hyperinflation of the chest and morbid obesity, and on mechanical ventilation, only one to two views can be obtained for evaluation. Cardiac function assessment, although limited, may still be possible in these situations through the remaining one to two views. If FoCUS reveals features of hypovolemia (as will be discussed later), a focused assessment with sonography for trauma (FAST) protocol (i.e., SIMPLE + FAST approach) is warranted to look for intra-abdominal bleeding and hemothorax. Although limited when compared to comprehensive echocardiography carried out in the cardiac laboratory, this approach provides valuable information concerning the pathology, heart function, and physiology to differentiate between different types of shock and guide subsequent management.

#### Chamber sizes

**Fig. 3:**
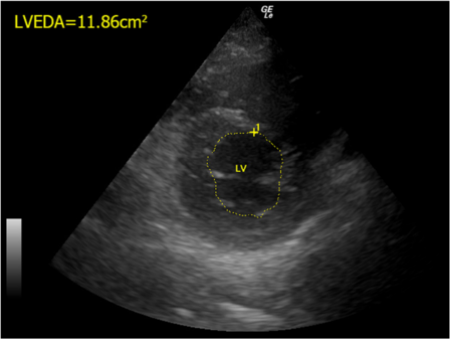
The normal LVEDA measurement. The LVEDA is measured at the level of mid-papillary level of left parasternal short axis view in a normal human being. (*LVEDA* left ventricular end diastolic area, *LV* left ventricle)

The size of the heart chambers reflects the preload status (i.e., the intravascular volume) and heart function based on the volume-pressure relationship of a compliant heart chamber, in the absence of pre-existing or concurrent diseases such as cardiomyopathy or massive myocardial infarction. LV end-diastole diameter (LVEDD) and LV end-diastole area (LVEDA) (Fig. 3) can be used to assess the circulatory volume status [20]. A LVEDA of less than 10 cm^2^ or a LVEDA index (LVEDA/body surface area) of less than 5.5 cm^2^/m^2^ indicates significant hypovolemia [21]. Obliteration of the LV cavity would be seen in severe hypovolemia [22]. In contrast, fluid overload can cause dilatation of the left ventricle. An LVEDA of more than 20 cm^2^ suggests volume overload [21]. However, in the case of severe RV dysfunction, LV can also be small due to underloading. Concentric LV hypertrophy and constrictive pericarditis may also lead to small LVEDA so caution should be taken for interpretation in these conditions.

The size of the RV can give clues to the right heart function. Normally, it should be smaller than the LV and the apex should be formed by LV, not RV (Fig. 1). It is easily visualized in the apical four-chamber view as a triangular-shaped structure. The normal basal diameter of RV should be less than 4 cm [23]. If it is enlarged acutely in the appropriate clinical setting, the diagnosis of acute right heart failure due to massive pulmonary embolism should be suspected. The end-diastole area ratio of RV/LV should be less than 0.6 in the normal heart. A ratio higher than 0.66 would suggest cor pulmonale [24]. When this happens together with a normal RV wall thickness (<5 mm in parasternal long views), then it is very likely that there is acute right heart failure resulting from massive pulmonary embolism.

#### IVS movement

**Fig. 4:**
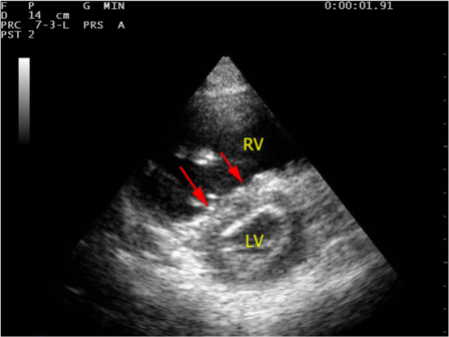
Acute cor pulmonale due to massive pulmonary embolism. A parasternal short axis view shows a dilated RV and D-shaped LV on parasternal view in a patient with massive pulmonary embolism. The flattened IVS is highlighted by *red arrows*. (*RV* right ventricle; *LV* left ventricle)

Normally, LV appears as a circular or donut-shaped structure (Fig. 1), and the IVS moves towards the center of LV during systole from the parasternal short axis view. When there is acute pulmonary embolism, high right ventricular pressure will cause more forceful and prolonged contracture of RV [24]. The IVS would then be pushed towards the left side, leading to flattening of the IVS or a D-shaped appearance of the LV on parasternal short axis view during end-systole or early-diastole (Fig. 4). In contrast, during systole, the LV contracts and the pressure in LV will become high again, pushing the IVS to the right side. This refers to “paradoxical movement” of the IVS.

#### IVC size and collapsibility

**Fig. 5:**
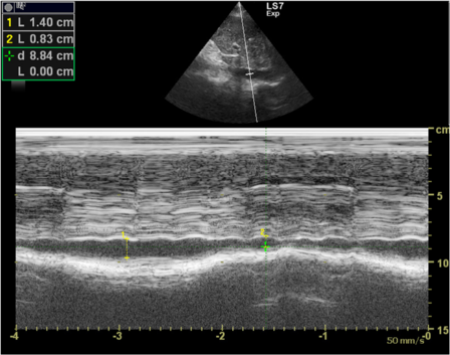
Normal M-mode tracing of the inferior vena cava throughout the respiratory cycle. Variation of the inferior vena cava diameter throughout the respiratory cycle is shown

Traditionally, the central venous pressure (CVP) has been used for fluid status assessment and monitoring. Nonetheless, it is not only invasive but also proven to correlate poorly with the blood volume status in a systematic review [25]. Recent studies suggest the use of echocardiography to assess fluid status and fluid responsiveness by measuring IVC size and its collapsibility [26–30]. The IVC can be seen at the subxyphoid area, slightly off midline to the right of the abdominal aorta on transverse view. IVC size should be measured in longitudinal view around 2 cm caudal to the point where the hepatic vein joins the IVC to the right atrium (RA) [31]. One should bear in mind that incorrect measurement will occur if the longitudinal view of IVC is off axis. M-mode tracing of the size of the IVC throughout the respiratory cycle can be obtained at this point (Fig. 5). In patients with spontaneous breathing effort, the IVC collapses on inspiration but distends on expiration due to change in intrathoracic pressure. The reverse occurs in patients on mechanical ventilation.

IVC size can be used as a surrogate measurement of preload and volume status and therefore right atrial pressure (RAP). IVC diameter can be used to estimate RA pressure. The American Society of Echocardiography suggested the cutoff value of 2.1 cm [32]. IVC diameter <2.1 cm that collapses >50 % with inspiration would correlate with RA pressure of 3 mmHg (range, 0–5 mm Hg) while an IVC diameter >2.1 cm that collapses <50 % with inspiration suggests high RAP of 15 mm Hg (range, 10–20 mmHg). In patients with hypovolemic shock, the IVC diameter will be expected to be <2.1 cm and collapse >50 % with inspiration. In a recent meta-analysis of data from five studies on the sonographic measurement of the IVC in assessing the fluid status in the emergency department (ED), it was evidenced that the maximum IVC diameter is lower (6.3 mm 95 % CI 6–6.5 mm) in patients with hypovolemia than euvolemia [28]. Resuscitation of hypotensive patients usually involves fluid challenge. IVC diameter may give us some clues. The IVC distensibility index where maximum IVC diameter minus minimal IVC diameter divided by minimal IVC diameter times 100 % was found to be useful in predicting fluid responsiveness using the cutoff of 18 % in mechanically ventilated patients [29]. However, for patients with spontaneous breathing, the value of IVC size was less distinguished in predicting fluid responsiveness. With the cutoff of 40 %, the IVC collapsibility index that is maximum IVC diameter minus minimum IVC diameter divided by maximum IVC diameter times 100 % would only give a sensitivity of 70 %, specificity of 80 %, positive predictive value of 72 %, and negative predictive value of 83 % [30]. In trauma patients with hemorrhage, IVC measurement in addition to FAST is also helpful for managing trauma patients with hypovolemia to guide fluid therapy and shorten the time to operation theater [33, 34].

#### Intimal flap in aortic dissection

**Fig. 6:**
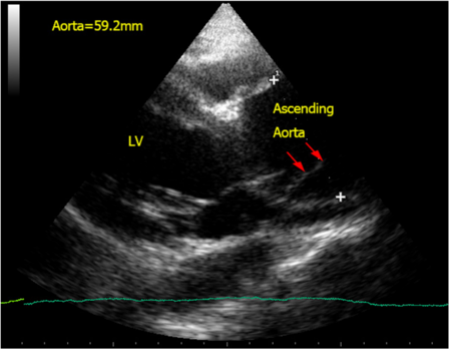
Aortic dissection. Intimal flap (*red arrow*) is seen in the dilated proximal ascending aorta (5.9 cm) in a confirmed case of type A aortic dissection. (*LV* left ventricle)

Aortic dissection is a potentially fatal but challenging vascular emergency. The cardinal feature of this disorder is a tear of the intimal layer of the aorta causing blood to dissect between layers of the aortic wall and propagate along the vessel. Hypotension was found to present in 16.4 % of patients with aortic dissection [35]. However, clinical features and chest X-ray findings are seldom confirmatory. With the availability of FoCUS, we can rule in aortic dissection and detect the associated complications, e.g., pericardial effusion, cardiac tamponade, pleural effusion, myocardial ischemia, and aortic regurgitation. The proximal part of the ascending aorta can be assessed by FoCUS from the parasternal long axis view. The abdominal aorta (as discussed later) should be assessed in suspected cases of aortic dissection. The pathognomonic sonographic feature of aortic dissection is the presence of intimal flap which appears as an echogenic thin linear structure separating the true and false lumens inside the aorta (Fig. 6). Dilatation of the aortic root (>4 cm), aortic regurgitation, and presence of pericardial effusion are auxiliary findings. The sensitivity and specificity of TTE for type A aortic dissection are 78–90 and 87–96 %, respectively [36, 37]. Positive findings can speed up management and confirm life-threatening complications. Since only the proximal part of the ascending aorta can be seen in TTE, aortic dissection cannot be ruled out completely and other modality of imaging should be considered in patients with high clinical suspicion.

#### Mass in cardiac chambers: thrombus/myxoma

**Fig. 7:**
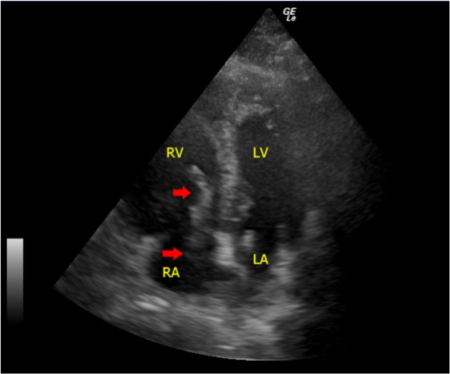
Pulmonary embolism. In this apical four-chamber view, echogenic blood clots (*red arrows*) in the right atrium protruding into the right ventricle through the tricuspid valve during diastole are seen in a patient with confirmed massive pulmonary embolism(*RA* right atrium, *RV* right ventricle, *LA* left atrium, *LV* left ventricle)

Intra-cardiac masses are not commonly seen on sonographic examination. But when present, in the appropriate clinical setting, they help establish the diagnosis of obstructive shock. The presence of intramural thrombus in right-sided cardiac chambers can confirm the clinical suspicion of pulmonary embolism and guide subsequent treatment [38]. Intracardiac thrombi appear as echogenic masses (Fig. 7) in the right atrium, right ventricle, pulmonary arteries, and IVC. The thrombi may be attached to the atrial or ventricular wall or be freely mobile [39]. LV thrombi resulting from causes such as atrial fibrillation, dilated left atrium (LA), and myocardial infarction may cause obstruction if large enough to occlude the left ventricular outflow tract (LVOT) and mitral valve. Another intracardiac mass that can cause obstructive shock is the atrial myxoma. It is the commonest primary cardiac tumor and most commonly involves the left atrium (75 %) [40]. It is often attached to the atrial wall and protrudes between the atrium and ventricle causing obstruction throughout the cardiac cycle, like a pinball machine. Occasionally, metastatic tumors may also cause obstructive shock in a similar manner as atrial myxoma [41].

#### Myocardium

During systole, different parts of the LV thicken in a coordinated fashion to act as a pump to eject blood out of the heart. According to the European society of Cardiology and American Society of Echocardiography, the LV can be divided into 17 segments and each individual segment can then be graded as normal/hyperkinetic, hypokinetic (reduced thickening), akinetic (absent thickening), or dyskinetic (abnormal thinning and stretching especially in aneurysm) according to their motions during systole [23, 42]. This can give clue to myocardial ischemia/infarct and the culprit coronary vessel involved when the areas of abnormal regional wall motion correspond to the territory supplied by the culprit vessel. With compatible sonographic findings and clinical picture, primary percutaneous intervention will be warranted when myocardial ischemia/infarct is believed to be the cause for cardiogenic shock.

Abnormal thickening of the myocardium (LV posterior wall and interventricular septum thickness >1 cm at end-diastole; RV free wall >5 mm) is suggestive of chronic heart conditions resulting from pressure overload (e.g., hypertensive cardiomyopathy, hypertrophic cardiomyopathy, pulmonary hypertension, and aortic stenosis). Together with gross dilatation of ventricle and atrium, detection of myocardial thickening is considered by the latest international consensus to be an essential part of FoCUS [13]. It can help avoiding misdiagnosing pre-existing heart conditions (e.g., chronic cor pulmonale) as an acute one (e.g., acute massive pulmonary embolism) and avoiding inappropriate treatments (e.g., intravenous fibrinolytic).

#### Pericardial effusion vs pleural effusion

**Fig. 8:**
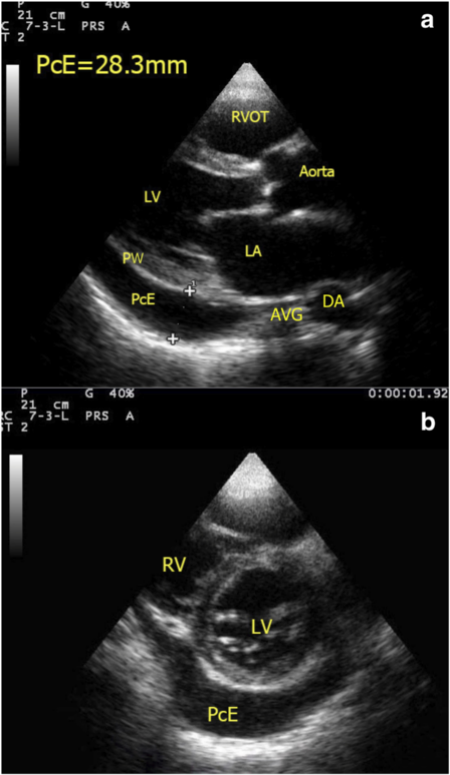
**a**, **b** Pericardial effusion (*PcE*). A large amount of pericardial effusion (*PcE*) is seen in both parasternal long axis and short axis view. The maximum size of the pericardial effusion measures 2.83 cm. Note the relationship between the pericardial effusion and the descending aorta (*DA*). (*RV* right ventricle, *LV* left ventricle, *PW* posterior wall of LV, *PcE* pericardial effusion, *LA* left atrium, *RVOT* right ventricular outflow tract, *DA* descending aorta, *AVG* atrioventricular groove)

**Fig. 9:**
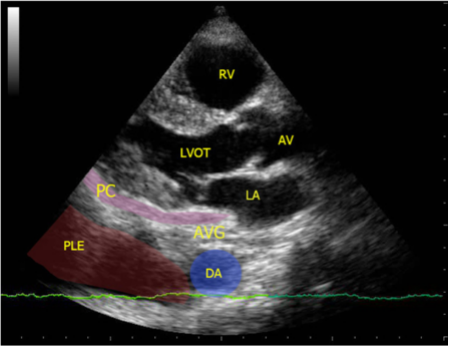
Pleural effusion (*PLE*). The parasternal long axis view shows anechoic pleural effusion (*PLE*) accumulated posterior to the descending aorta (*DA*). Pericardium (*PC*) is represented by the *pink strip* here. Also pleural effusion, if large amount, can extend beyond the atrioventricular groove in contrast with pericardial effusion which terminates at the atrioventricular groove. (*AV* aortic valve, *LVOT* left ventricular outflow tract, *PC* pericardium, *PLE* pleural effusion, *RA* right atrium, *RV* right ventricle, *DA* descending aorta, *AVG* atrioventricular groove)

The pericardial sac is a potential space for fluid to collect due to both systemic illness (e.g., connective tissue disease and uremia) and local pathology (e.g., myocardial rupture, aortic dissection, and metastasis). Although difficult to detect by physical exam or chest X-ray, pericardial effusion is easily picked up by FoCUS. Usually, pericardial effusion appears as an anechoic rim surrounding the heart, best seen in the parasternal long axis view or subxyphoid four-chamber view. However, if the effusion is caused by inflammatory condition or hemorrhage (i.e., hemopericardium), there may be echogenicity within the pericardial sac. The pericardium appears as a densely echogenic film-like reflection posterior to the anechoic pericardial effusion (Fig. 8a, b). Sometimes pleural effusion can also be detected by echocardiography, and novice sonographers may find it confusing. Pericardial effusion can be differentiated from pleural effusion as pericardial effusion is located anterior to the descending aorta and does not extend beyond the atrioventricular groove (Fig. 9). Sometimes, pericardial or epicardial fat can also be mistaken as pericardial effusion. Epicardial fat usually appears as an echogenic structure lying within the pericardial sac just anterior to the heart on the parasternal long axis view [43]. The size of pericardial effusion should be quantified according to its maximum thickness measured during diastole (small <1 cm not circumferential, moderate <1 cm circumferential around the heart, large 1–2 cm circumferential, very large >2 cm with/without evidence of cardiac tamponade) [44]. Acute accumulation of pericardial effusion can lead to impaired right heart filling and, in turn, cardiac tamponade that will be discussed in the next session.

#### LV systolic function

Echocardiography or cardiac ultrasound can give an accurate assessment of global function of the left ventricle and guide subsequent treatment (e.g., inotropic support versus fluid therapy). There are several options to assess the LV systolic function sonographically, including fractional shortening (FS) and LV ejection fraction (LVEF).

In M-mode, FS of the left ventricle can be assessed by placing a cursor just near the tip of the mitral valve leaflets in parasternal long axis view. The M-mode tracing will show the change in LV diameter during the cardiac cycle and FS can be calculated by the following formula:

$$ \mathrm{F}\mathrm{S} = \left(\mathrm{LVEDD}-\mathrm{LVESD}\right)/\mathrm{LVEDD} \times 100\% $$

The normal value should be 25–45 % for adults [42]. If the value falls below <15 %, severe LV systolic dysfunction is present. This measurement is very simple and easy. However, the measurement must be done perpendicular to the axis of the left ventricle, and the ventricle should not be foreshortened. There is also an assumption of no severe dysfunction in other parts of the left ventricle.

In B-mode, the LVEF can be measured by the modified Simpson biplane method. Most modern ultrasound machines have the calculation package pre-installed. The endocardial margins of the LV are traced in systole and diastole from two different views (i.e., two individual planes perpendicular to each other) to calculate the volume change between systole and diastole. The normal LVEF should be >55 %, and <30 % indicates severe left ventricular systolic dysfunction [42]. This is not a simple method compared with the FS, and the endocardial margins have to be traced correctly or under/overestimation of the LVEF may result. In emergency settings, suboptimal images of the LV and inadequate cardiac views would render this method less practical.

Assessment of LVEF by eyeballing appears the most feasible yet reliable method for estimating the LV systolic function. It can be done through assessing the movement and thickening of the LV myocardium, the change in size and shape of the LV chamber as well as the mitral valve anterior leaflet excursion in the cardiac cycle. It was found that the accuracy of eyeballing estimation correlated well with other quantitative methods including Simpson biplane ejection fraction, fractional shortening, wall motion score index, and aortic valve (AV) plane displacement [45, 46]. This advantage is not confined only to the experienced cardiologists. With focused training, the estimation of LV ejection fraction by emergency physicians had a strong agreement with cardiologists [47–49]. Even inexperienced emergency medicine trainees could achieve good agreement on the visual estimation of LV ejection fraction with cardiologists after web-based learning and proctored practical training (*K* = 0.79, 95 % CI 0.773 to 0.842) [49]. Thus, visual estimation of the LV ejection fraction should form an important part of left heart systolic function assessment in FoCUS, in particular when quantitative measurements are not possible due to poor echogenicity of the heart and limited cardiac views in some patients.

#### Abdominal aortic at the epigastrium

**Fig. 10:**
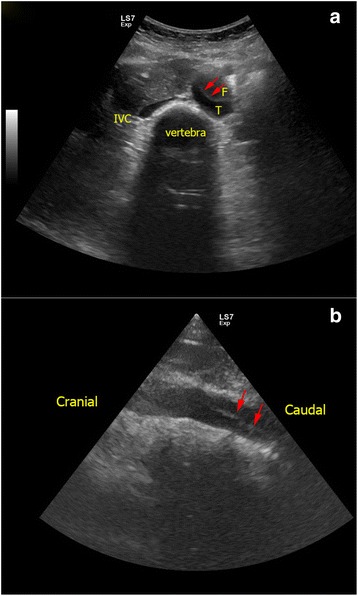
**a**, **b** Aortic dissection of abdominal aorta. In this patient with extensive Stanford type A aortic dissection, intimal flap (*red arrows*) is seen inside the lumen of abdominal aorta as an echogenic film separating the false lumen (*F*) and true lumen (*T*). (*F* false lumen, *T* true lumen, *IVC* inferior vena cava)

The proximal part of abdominal aorta can be easily visualized by ultrasound. It should be integrated into the scanning protocol of FoCUS in addition to the IVC measurement. It lies along the mid-line of the abdomen, on the left side of the IVC, and anterior to the bony vertebra. The normal size of the abdominal aorta should be less than 3 cm. Abdominal aortic aneurysm (AAA) is diagnosed when the diameter is >3 cm. AAA can rupture and cause profound hypotension due to hypovolemia. Clinical presentation may be subtle, and reliance on physical findings alone may miss this potentially fatal condition as the sensitivity of abdominal palpation is only 68 % [42, 50]. PoCUS has outstanding sensitivity and specificity for AAA approaching 100 % and has been proved to shorten the time to emergency operation [51, 52]. Other arterial catastrophes including rupture of splenic artery aneurysm can also be detected in a similar way to AAA [53]. Echogenic intimal flap seen inside the aortic lumen can confirm the diagnosis of aortic dissection (Fig. 10a, b). This may improve the sensitivity of FoCUS in diagnosing aortic dissection, especially in cases involving the descending aorta.

### Evaluation of undifferentiated shock by SIMPLE approach

#### Hypovolemic shock

**Fig. 11:**
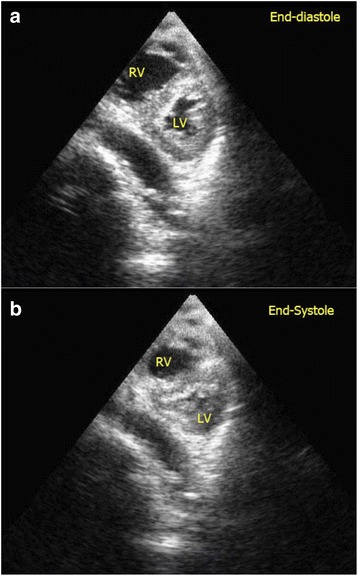
**a**, **b** Severe hypovolemic shock. Kissing walls of left ventricle on parasternal short axis view is shown. The left ventricle is obliterated during systole. This patient suffered from severe hypovolemia due to gastrointestinal bleeding. (*RV* right ventricle, *LV* left ventricle)

**Fig. 12:**
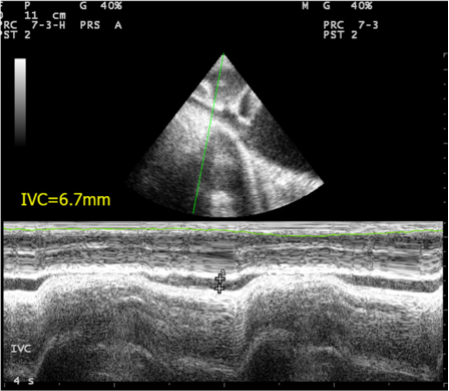
Collapsed IVC. IVC thickness is markedly reduced (thickness = 6.7 mm) with complete collapse on inspiration in a patient with hypovolemic shock

In patients with hypovolemia, the left ventricle becomes small with a smaller LVEDA (<10 cm^2^). The lumen of the LV may even be obliterated and the ventricular walls are seen to be “kissing” (Fig. 11a, b) [22]. The IVC collapses, and the size becomes less than 2 cm with >50 % collapsibility (Fig. 12). Hyperdynamic LV with normal or higher than normal ejection fraction and normal myocardial thickening is found. Depending on the source of bleeding, hemothorax may be an incidental finding in FoCUS but it should not be misinterpreted as pericardial effusion. The epigastric area should be screened for the presence of an aortic aneurysm. If an aortic aneurysm is found in a hypotensive patient, aneurysmal rupture should be suspected and urgent surgical consultation is warranted. FAST scan should also be done when no obvious sources of bleeding can be identified in the context of hypovolemic shock. Fluid responsiveness can be predicted by using the IVC collapsibility index in ventilated patients to guide subsequent fluid therapy.

#### Cardiogenic shock

**Fig. 13:**
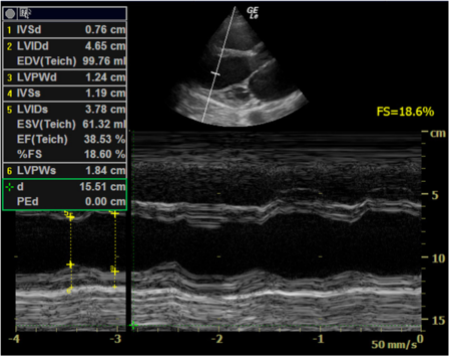
Poor LV systolic function. This parasternal long axis view shows a dilated LV with poor fractional shortening (FS = 18.6 %) in a patient with dilated cardiomyopathy and hypotension (*FS* = fractional shortening)

Echocardiography or cardiac ultrasound definitely has a role in managing cardiogenic shock due to LV dysfunction and valvular dysfunction. LV would be dilated, and fractional shortening would be impaired (Fig. 13). Dilated IVC >2.1 cm with the absence of respiratory variability is expected. Regional wall motion abnormality may be seen if the underlying cause for cardiogenic shock is myocardial ischemia. The whole myocardium would be hypokinetic in the case of global systolic dysfunction (e.g., due to myocarditis). As mentioned before, LV systolic function can be assessed by quantitative measurements including FS and LVEF by using the modified Simpson biplane method. However, all these measurements require good visualization of the LV and clear delineation of the endocardium. Errors commonly occur when the optimal image of the LV cannot be obtained in emergency settings. Thus, visual estimation, so called eyeballing, would be more practical in these situations. By obtaining valuable information on pump function, inotropic support and judicious fluid administration is indicated. Emergency transferal of the patients to cardiac catheterization facility for revascularization can also be facilitated in myocardial infarction.

## Obstructive shock

### Cardiac tamponade

**Fig. 14:**
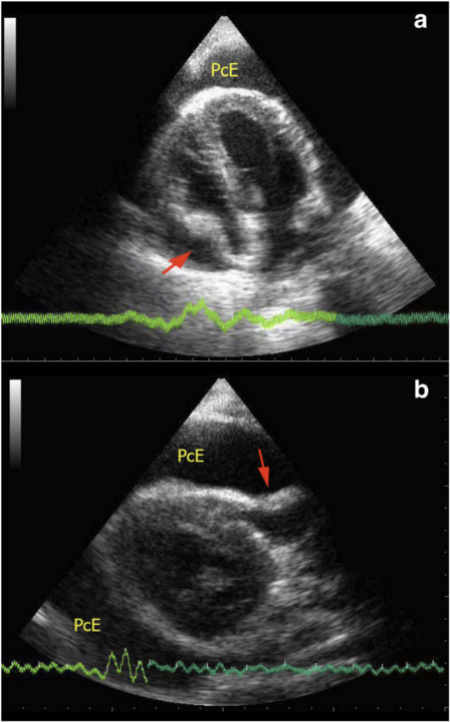
**a**, **b** Cardiac tamponade. These two images show collapsed RA and RV (*red arrows*) on apical four-chamber view and parasternal long axis view in a patient with cardiac tamponade (*PcE* = pericardial effusion)

One of the major causes for obstructive shock is cardiac tamponade. The presence of pericardial effusion and hypotension raises the suspicion of cardiac tamponade. Large amounts of effusion would cause cardiac tamponade, but even small effusions, if accumulated rapidly, can cause cardiac tamponade due to the tough, non-distensible nature of the pericardial sac. Although cardiac tamponade is essentially a clinical diagnosis, FoCUS can help to confirm the presence of pericardial effusion, provide useful real-time hemodynamic information and tamponade physiology, and guide therapeutic pericardiocentesis. Sonographic features of cardiac tamponade include RA collapse, RV diastolic collapse, distended IVC, and respiratory variation of the mitral inflow velocity. The latter is essentially the sonographic version of pulsus paradoxicus. Pulsed wave Doppler is required to detect the variation so it is out of the scope of this SIMPLE approach (Table 2). RA collapse can be easily recognized when the RA inverts during ventricular end-diastole when the pressure inside the atrium becomes lowest (Fig. 14a). RV diastolic collapse is recognized as part of the RV free wall not expanding during early diastole (Fig. 14b). RA collapse is a more sensitive but non-specific sonographic sign of cardiac tamponade while diastolic RV collapse is considered to be more specific. Plethoric IVC without any variation during respiration is an additional sign to look for in cardiac tamponade. When the diagnosis of cardiac tamponade is established, FoCUS-guided pericardiocentesis can then be performed. Ultrasound-guided pericardiocentesis is now considered to be the standard of care because it carries higher successful rate and fewer complications than the blind approach [54, 55]. The apex was identified as the optimal location for pericardiocentesis in 1127 consecutive patients from the Mayo Clinic over 21 years [56].

### Massive pulmonary embolism

**Fig. 15:**
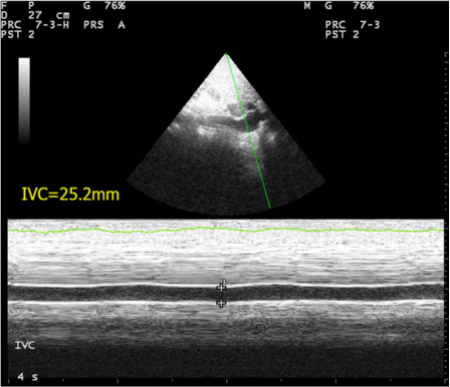
Distended IVC. This is the M-mode tracing of IVC in a patient with massive PE. The IVC is plethoric of a diameter >2.1 cm with only minimal respiratory variation (*IVC* = inferior vena cava)

Massive pulmonary embolism causes acute RV dysfunction. RV is usually dilated with basal diameter >4 cm and RV/LV ratio >0.6. The normal triangular-shaped RV is distorted, and the apex is no longer dominated by the LV on the apical four-chamber view. The IVS shows paradoxical movement, and D-shaped LV chamber would be appreciated in the parasternal short axis view (Fig. 4). The IVC is distended with minimal or absent respiratory variation (Fig. 15). Free flowing echogenic thrombus may occasionally be seen in the right heart and IVC (Fig. 7). McConnell’s sign which is defined as mid-RV free wall akinesia with sparing of the apex may occasionally be seen [57]. It is thought to be a specific but not very sensitive sign for acute pulmonary embolism (sensitivity 77 %; specificity 96 %). This specific sign is believed to be caused by tethering of the RV to the hyperdynamic LV apex [57, 58]. LV becomes hyperdynamic as the left heart is trying hard to compensate for the hypotension. The American College of Chest Physicians suggests that fibrinolysis should be warranted in patients with hypotension due to massive pulmonary embolism [59]. When severe right heart dysfunction is confirmed by FoCUS with hypoxia, hypotension, and tachycardia, the diagnosis of massive pulmonary embolism should rank top on the list of differentials. Intravenous fibrinolytic therapy should be prudently considered if there is no contraindication to reverse acute right heart dysfunction due to pulmonary embolism and surgical embolectomy may be needed in patients with contraindication to systemic fibrinolysis.

### Aortic dissection

**Fig. 16:**
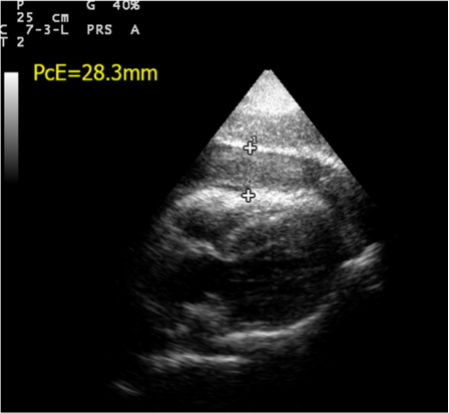
Hemopericardium and cardiac tamponade. This subxyphoid four-chamber view shows echogenic clots and hemopericardium in a patient with cardiac tamponade due to aortic dissection (*PcE* pericardial effusion; thickness = 28.3 mm)

Aortic dissection can be picked up by FoCUS when the intimal flap is in the aorta (Fig. 6). The complications of aortic dissection can also be detected. Retrograde dissection into the pericardial sac can cause pericardial effusion and even cardiac tamponade. Echogenic pericardial effusion and even clots are occasionally seen (Fig. 16). The IVC becomes plethoric when cardiac tamponade is present. Regional wall motion abnormality may also be detected in the case of acute myocardial ischemia secondary to ostial occlusion by the intimal flap, usually involving the right coronary artery [60]. It is often rewarding to scan the abdominal aorta in patients suspicious of distal aortic dissection as sometimes the intimal flap which cannot be seen in the ascending aorta may be seen here and the diagnosis is obvious (Fig. 10a, b).

#### Septic shock

Septic shock represents a distinctive spectrum of hemodynamic instability. In the early stage, the afterload is reduced and left ventricular dysfunction, although present, is masked by the severely reduced afterload due to sepsis [61, 62]. Thus, FoCUS will find a normal LVEDA but small left ventricular end-systolic area (LVESA) and a hyperdynamic LV. There is a substantial decrease in the size of the LV from diastole to systole in contrast to hypovolemia where the LV size is small throughout the cardiac cycle. The IVC would collapse in this stage with >50 % inspiratory collapse. After the initial phase, myocardial depression occurs in around 60 % septic patients [63]. Once the afterload is restored by vasopressors and fluid therapy, the LV myocardial dysfunction is unmasked. LV would be normal or dilated with myocardial hypocontractility [61–63]. At this stage, the IVC is distended and the respiratory collapsibility is lost similar to the profile in cardiogenic shock. Recognizing these sonographic findings can help clinicians tailoring appropriate treatments to different stages of septic shock.

### SIMPLE approach versus other protocols

Since 2001, different protocols for shock assessment have been described in the literature. Table 3 summarizes and compares the current major protocols for undifferentiated shock and cardiac arrest. There is a growing trend towards integrating different aspects of point-of-care ultrasound including focused cardiac ultrasound, IVC and aorta assessment, and lung scan into different protocols [9, 70, 75–77]. The goal is to find a systematic and practical way to classify the challenging but non-specific clinical syndrome of circulatory failure into four more specific and manageable types of shock.

**Table 3:** Summary of current major protocols of point-of-care ultrasound for undifferentiated shock/cardiac arrest

Protocols	Year of publication	Cardiac views	IVC size	Abdominal aorta	Lung scan	Remarks
UHP [11]	2001	SXP+/−PLX, PSX	No	Yes	No	Simplified version of extended FAST
Trinity [64]	2002	PLX, PSX	No	Yes	Pleural effusion only	Similar to extended FAST
FATE [65]	2004	PLX, PSX, AP4, SXP	No	No	Pleural effusion only	Chronic pathologies included
BLEEP [66]	2004	SXP, PSX	Yes	No	No	Pediatric patients only
CAUSE [67]	2007	4 views	Yes	Yes	Yes	Cardiac arrest
FEER [68]	2007	SXP, PLX, PSX, AP4	No	No	No	Integrated into ACLS protocol for cardiac arrest
BEAT [69]	2008	PLX, PSX, AP4, SXP	Yes	No	No	Surgical patients; SV and CI included
ACES [10]	2008	SXP, PLX, AP4	Yes	Yes	Pleural effusion only	
RUSH-HIMAP [70]	2009	PLX, AP4	Yes	Yes	Yes	
RUSH-pump, pipe, tank [9]	2010	PLX, PSX, SXP, AP4	Yes	Yes	Yes	Physiological model of pump, pipe, tank
FEEL [71]	2010	SXP, PLX. AP4 (any one of them)	No	No	Pleural effusion only	Cardiac arrest and peri-arrest state
EGLS [72]	2011	PLX, PSX, AP4, SXP	Yes	No	Yes	Lung scan first approach
FREE [73]	2011	PLX, PSX, AP4, SXP	Yes	No	No	Trauma patients
FALLS [74]	2012	Not specifically mentioned	No	No	Yes	Mainly lung scan
FAST and RELIABLE [75]	2012	PLX, PSX, AP4, SXP	Yes	Yes	Yes	Ectopic pregnancy included
Volpicelli et al. [76]	2013	PLS, SXP, AP4	Yes	Yes	Yes	Similar to RUSH [9]
Shokoohi et al. [77]	2015	SXP, PLX, PSX, AP4	Yes	Yes	Yes	FAST included
SIMPLE	2016	PLX, PSX, AP4, AP2, SXP	Yes	Yes	No	Easy-to-remember checklist of sonographic findings; intracardiac mass and intimal flap included; can be combined with FAST

*PSX* parasternal short axis view, *PLX* parasternal long axis view, *SXP* subxyphoid view, *AP4* apical four-chamber view, *AP2* apical two-chamber view, *IVC* inferior vena cava, *ACLS* advanced cardiac life support, *ICU* intensive care unit, *FAST* focused assessment with sonography for trauma, *SV* stroke volume, *CI* cardiac index

In contrast to existing protocols like rapid ultrasound for shock and hypotension (RUSH) [9], abdominal and cardiac evaluation with sonography in shock (ACES) [10], undifferentiated hypotension patient (UHP) [11], Trinity [64], or focused assessed transthoracic echocardiography (FATE) [65], every single letter in the SIMPLE approach represents a specific assessment in cardiac ultrasound. This simple mnemonic provides clinicians with a simple and easy-to-remember, yet valuable checklist of sonographic findings to look for when managing patients in shock. Unlike the RUSH protocol, physiology is not emphasized in the SIMPLE approach but systematic interpretation of sonographic findings can help the clinicians narrow down the differential diagnosis of shock and guide initial therapy (e.g., small and kissing LV with flat IVC already warrants fluid resuscitation and hypovolemia is suspected, while dilated and hypokinetic LV would suggest cardiogenic shock and inotropic support is needed). It can also help avoiding complications associated with indiscriminate use of fluid therapy and inotropes.

To my knowledge, this is the first protocol to include two specific findings explicitly: intramural mass and intimate flap so as to improve the diagnostic power of two challenging and lethal conditions, namely massive pulmonary embolism and aortic dissection. Although abdominal assessment is not routinely included in the SIMPLE approach, combination with FAST to look for the source of intra-abdominal bleeding is indicated when FoCUS reveals features of hypovolemia. As most emergency physicians and intensivists have been using FAST scan routinely in trauma assessment, a combination of SIMPLE with FAST (SIMPLE + FAST) would easily be incorporated into their daily practices. SIMPLE + FAST suggests cardiac assessment first before abdominal assessment in order to detect obstructive and cardiogenic shock, in contrast to the FAST + RELIABLE protocol suggested by Liteplo et al. [75]. This allows early specific treatment such as pericardiocentesis and inotropes to correct the circulatory failure and prevent indiscriminative fluid challenge which is detrimental in cardiogenic shock.

### Current evidence of FoCUS for evaluation of shock

Among the literature, there is growing evidence demonstrating that FoCUS could improve the diagnostic accuracy and change the clinical management. Although the results of this evidence may not necessarily prove that FoCUS can lead to better patient survival and shorten the hospital stay, it can logically be assumed that with more accurate diagnostic capability for various types of shock, implementation of FoCUS could lead to a better clinical outcome in patients with circulatory failure.

FoCUS is helpful in confirming the correct diagnoses and detecting the etiology of shock. In a randomized trial in 184 patients by Jones et al. in the emergency department, early goal-directed ultrasound at 0 min was found to correctly diagnose the etiology of shock in 80 % of patients compared 50 % of patients in the group only received standard care at the initial 15 min of presentation in the emergency department [78]. This trial can be concluded into two important points. Firstly, it was the first study to prove that early focused ultrasound can allow the emergency physicians to narrow the differential diagnoses of shock. Secondly, it proved that early focused ultrasound at the initial presentation is feasible and can be combined with standard care interventions, e.g., venous access establishment, electrocardiography, blood sample analysis, and chest radiography.

Subsequent trials on protocol-driven ultrasound for diagnosis of shock further confirmed the role of FoCUS to diagnose and differentiate different types of shock in the emergency department. Volpicelli et al. did a prospective study on 108 ED patients in undifferentiated shock by comparing the sonographic diagnosis with the final clinical diagnosis [76]. The ultrasound assessment in this study included FoCUS and IVC assessment, lung scan, abdominal scan for free fluid, and leg scan for deep vein thrombosis. They found a very good concordance between the ultrasound diagnosis and the final clinical diagnosis (*k* = 0.710). Ghane et al. also found similar finding in a study in 52 ED patients by using RUSH protocol (*k* = 0.7) [79]. It was also found that ultrasound achieved 100 % sensitivity for hypovolemic and obstructive shock, 91.7 % sensitivity for cardiogenic shock, and 94.6–100 % specificity for all types of shock. However, in distributive and mixed type of shock, the sensitivity was found to be lower only 70–75 %. The same group also found similar results in another study on 77 patients [80]. The common limitation of the above three studies is that ultrasound assessments were done by either radiologist or emergency physicians experienced in PoCUS, and so the results may not be generalizable to other inexperienced clinicians from other specialties.

Apart from correct diagnosis and differentiation of shock, the other major role of FocUS for shock is to tailor the treatment according to the underlying etiology and improve the clinical outcome of patients. In an observational study conducted on 220 patients in intensive care unit, use of FoCUS by hand-held ultrasound device was found to be associated with significantly lower fluid prescription (49 vs 66 mL/kg, *p* = 0.01) and more dobutamine use (22 vs 12 %, *p* = 0.01) than the historical control group which was managed in a standard fashion [81]. More importantly, this study found that FoCUS group had better 28-day survival (66 vs 56 %, *p* = 0.04) and a reduction in acute kidney injury (20 vs 39 %). These findings are supportive of using FoCUS to guide the resuscitation of hypotensive patients. The limitations of this study include no randomization, use of historical control, and a significant number of patients (14 %) with significant valvular pathologies. Another recent study performed in ED also confirmed the impact of FoCUS findings on management plan (in 24.6 % of patients), including the use of intravenous fluid, vasoactive agents, or blood products. This study also found an excellent concordance of protocol-driven ultrasound diagnostic protocol in undifferentiated hypotension with the final diagnosis (*k* = 0.80). Moreover, ultrasound was also found to influence the diagnostic imaging, consultation, and patient disposition in this study. Again, the limitations are the lack of randomization and use of single ultrasound operator.

## Conclusions

Managing patients in profound shock poses a very great challenge to clinicians. Correct diagnosis and timely specific treatment to restore the otherwise jeopardized circulation are vital to the survival of hypotensive patients. Throughout the past 10 years, FoCUS has emerged as one of the important allies of emergency physicians and intensivists to provide crucial answers to challenging clinical conditions. In properly trained hands, FoCUS can provide real-time valuable information on the pathology and physiology of circulation to differentiate between different types of shocks. Through the suggested SIMPLE approach, different types of shocks can be characterized according to 2D ultrasound findings and simple measurements (Table 2). This approach is not only simple and practical but also provides an easy-to-remember checklist of ultrasound findings for clinicians to focus on when managing patients with undifferentiated shock. Integrating SIMPLE approach with FAST scan (i.e., SIMPLE + FAST) can be feasible and particularly helpful in identifying intraperitoneal bleeding and initiating fluid resuscitation in hypovolemic shock. Current evidence supports the role of FoCUS in undifferentiated shock to improve the diagnostic accuracy, narrow the possible differential diagnoses, and guide specific management. More high-quality clinical trials are warranted to further look into the impact of FoCUS on the clinical outcomes, patient survival, and financial implication in future.

## Abbreviations

AAA, abdominal aortic aneurysm; ACES, abdominal and cardiac evaluation with sonography in shock [10]; ACLS, advanced cardiac life support; AF, atrial fibrillation; AMI, acute myocardial infarction; AP2, apical two-chamber view; AP4, apical four-chamber view; AV, aortic valve; AVG, atrioventricular groove; BEAT, bedside echocardiographic assessment in trauma/critical care [69]; BLEEP, bedside limited echocardiography by emergency physician [66]; CAUSE, cardiac arrest ultrasound exam [67]; CI, cardiac index; CVP, central venous pressure; DA, descending aorta; ED, emergency department; EGLS, echo-guided life support [72]; FALLS, fluid administration limited by lung sonography [74]; FAST, focused assessment with sonography for trauma; FATE, focused assessed transthoracic echocardiography [12]; FEEL, focused echocardiographic evaluation in life support and peri-resuscitation of emergency patients [71]; FEER, focused echocardiographic evaluation in resuscitation [73]; FoCUS, focused cardiac ultrasound; FS, fractional shortening; ICU, intensive care unit; IVC, inferior vena cava; IVS, interventricular septum; LA, left atrium; LV, left ventricle; LVEDA, left ventricle end-diastole area; LVEDD, left ventricle end-diastole diameter; LVEF, left ventricular ejection fraction; LVESA, left ventricular end-systolic area; LVOT, left ventricular outflow tract; PC, pericardium; PcE, pericardial effusion; PLE, pleural effusion; PLX, parasternal long axis view; PoCUS, point-of-care ultrasound; PSX, parasternal short axis view; PW, posterior wall of left ventricle; RA, right atrium; RAP, right atrial pressure; RUSH, rapid ultrasound for shock and hypotension [9]; RUSH-HIMAP, rapid ultrasound for shock and hypotension-heart, inferior vena cava, Morrison pouch with FAST exam view and hemothorax windows, aorta, and pneumothorax [70]; RV, right ventricle; RVOT, right ventricular outflow tract; SV, stroke volume; SXP, subxyphoid view; TTE, transthoracic echocardiography; UHP, undifferentiated hypotension patient [11]; WINFOUCS, World Interactive Network Focused on Critical UltraSound
